# Development and validation of a new diagnostic prediction model of ENHO and NOX4 for early diagnosis of systemic sclerosis

**DOI:** 10.3389/fimmu.2024.1273559

**Published:** 2024-01-29

**Authors:** Leting Zheng, Qiulin Wu, Shuyuan Chen, Jing Wen, Fei Dong, Ningqin Meng, Wen Zeng, Cheng Zhao, Xiaoning Zhong

**Affiliations:** ^1^ Department of Rheumatology and Clinical Immunology, The First Affiliated Hospital of Guangxi Medical University, Nanning, China; ^2^ Department of General Surgery, The Second Affiliated Hospital of Guangxi Medical University, Nanning, China; ^3^ Department of Respiratory and Critical Care Medicine, the First Affiliated Hospital of Guangxi Medical University, Nanning, China

**Keywords:** systemic sclerosis, prediction model, machine learning, ENHO, NOX4, macrophage

## Abstract

**Objective:**

Systemic sclerosis (SSc) is a chronic autoimmune disease characterized by fibrosis. The challenge of early diagnosis, along with the lack of effective treatments for fibrosis, contribute to poor therapeutic outcomes and high mortality of SSc. Therefore, there is an urgent need to identify suitable biomarkers for early diagnosis of SSc.

**Methods:**

Three skin gene expression datasets of SSc patients and healthy controls were downloaded from Gene Expression Omnibus (GEO) database (GSE130955, GSE58095, and GSE181549). GSE130955 (48 early diffuse cutaneous SSc and 33 controls) were utilized to screen differentially expressed genes (DEGs) between SSc and normal skin samples. Least absolute shrinkage and selection operator (LASSO) regression and support vector machine recursive feature elimination (SVM-RFE) were performed to identify diagnostic genes and construct a diagnostic prediction model. The results were further validated in GSE58095 (61 SSc and 36 controls) and GSE181549 (113 SSc and 44 controls) datasets. Receiver operating characteristic (ROC) curves were applied for assessing the level of diagnostic ability. Reverse transcription-quantitative polymerase chain reaction (RT-qPCR) was used to verify the diagnostic genes in skin tissues of out cohort (10 SSc and 5 controls). Immune infiltration analysis were performed using CIBERSORT algorithm.

**Results:**

A total of 200 DEGs were identified between SSc and normal skin samples. Functional enrichment analysis revealed that these DEGs may be involved in the pathogenesis of SSc, such as extracellular matrix remodeling, cell-cell interactions, and metabolism. Subsequently, two critical genes (ENHO and NOX4) were identified by LASSO and SVM-RFE. ENHO was found down-regulated while NOX4 was up-regulated in skin of SSc patients and their expression levels were validated by above three datasets and our cohort. Notably, these differential expressions were more pronounced in patients with diffuse cutaneous SSc than in those with limited cutaneous SSc. Next, we developed a novel diagnostic model for SSc using ENHO and NOX4, which demonstrated strong predictive power in above three cohorts and in our own cohort. Furthermore, immune infiltration analysis revealed dysregulated levels of various immune cell subtypes within early SSc skin specimens, and a negative correlation was observed between the levels of ENHO and Macrophages M1 and M2, while a positive correlation was observed between the levels of NOX4 and Macrophages M1 and M2.

**Conclusion:**

This study identified ENHO and NOX4 as novel biomarkers that can be serve as a diagnostic prediction model for early detection of SSc and play a potential role in the pathogenesis of the disease.

## Introduction

Systemic sclerosis (SSc) is a chronic connective tissue disease characterized by the fibrosis of skin and visceral organ affecting various internal organs such as the lungs, hearts, gastrointestinal tract and kidneys. Subset of SSc includes diffuse cutaneous SSc (dcSSc) and limited cutaneous SSc (lcSSc) (1). The global prevalence of SSc ranges from approximately 5 to 50 cases per 100,000 individuals (2). Despite its relatively low prevalence, due to no effective treatment available, SSc exhibits the highest mortality rate among all connective tissue diseases, with a five-year survival rate of 74.9%, which plummets to 40% in patients with concurrent visceral involvement, with pulmonary hypertension and pulmonary fibrosis being the main causes of death (3, 4). The pathogenesis of SSc remains unknown, however, potential mechanisms include immune dysregulation, vasculopathy and excessive production and deposition of extracellular matrix (5). Notably, immune dysregulation and inflammation serve as the initiating and central factor in early stage of SSc (6–9). Abnormal activation of various immune cells, such as dendritic cells, macrophages, T and B lymphocytes, along with the secretion of diverse cytokines and autoantibodies contribute to the over-activation of fibroblasts. This process leads to increased synthesis of extracellular matrix and subsequent fibrosis of skin and visceral organs. Since fibrosis is largely untreatable, it is mandatory to start the treatment before fibrosis develops.

Different from advanced SSc, which mainly presents fibrosis, early SSc is characterized by inflammation and is considered to be the window of opportunity for effective treatment of SSc (10). For example, of the 7 patients treated with mycophenolate mofetil, all 4 patients with improved skin were in the inflammatory subgroup, and those identified as fibroproliferative subgroup before treatment did not have improvement (11). Meanwhile, four of the five dcSSc patients who had an improvement with abatacept treatment were in the inflammatory subgroup at the baseline (12). Whether patients can be treated in time before collagen deposition occurs, the key is early diagnosis of SSc. However, the diagnosis of early SSc remains a challenge for doctors (13). SSc has broad clinical heterogeneity, ranging from a milder subset to a more rapidly extensive fibrosis of skin and visceral organs. Additionally, most of the typical signs and symptoms are absent in early stage SSc. The American Rheumatism Association(ARA) preliminary criteria (1980) for SSc were ill-suited to identify an early SSc and the limited subset (14). The new 2013 ACR/EULAR classification criteria for SSc presents a higher specificity and sensitivity compared to the 1980 ARA criteria, however, those with a higher risk of developing SSc, characterized by Raynaud’s phenomenon(RP), puffy fingers, specific autoantibodies, and/or capillaroscopic specific abnormalities, could not achieve a score sufficient to be classified as SSc (15, 16). Therefore, even new 2013 ACR/EULAR classification criteria may lead to a possible late recognition of SSc. Most SSc patients present with RP as the initial symptom, however, RP is widely diffuse in the population, and 90% of cases are idiopathic (17, 18). Moreover, RP is not exclusive to SSc, as it is also seen in other connective tissue diseases (19). In addition, early identification and diagnosis of SSc are also important for drug clinical trials, which can help select appropriate SSc patients for therapeutic clinical trials and improve the efficacy of trials. Whereas the optimal design of clinical trials to test new therapeutics for SSc has suffered from difficulties in patient selection (20). Therefore, the need for early diagnosis of SSc is largely unmet.

Transcriptomics investigations entail the comprehensive profiling of all transcripts within a tissue or cell, thereby unveiling alterations in gene expression specific to certain disease states. By employing high-throughput sequencing technologies such as RNA-seq, researchers can acquire gene expression data spanning the entire genome and conduct differential gene expression analyses to pinpoint disease-associated markers (21, 22). These differentially expressed genes offer valuable insights into disease mechanisms and hold potential as diagnostic and therapeutic targets. Given the escalating magnitude of bioinformatics data, the integration of machine learning and artificial intelligence has gained increasing prominence in the identification of disease biomarkers (23, 24). These methodologies possess the capacity to effectively handle vast amounts of genomic data and unveil. intricate patterns and associations. Notably, machine learning algorithms have been employed to construct prediction models that leverage genomic data and clinical features, thereby aiding in disease diagnosis and treatment decision-making (25). Recent investigations have utilized machine learning techniques to identify novel biomarkers for various diseases including SSc. However, the application of machine learning methods to construct diagnostic prediction models specific to SSc is currently rarely reported (26, 27). Furthermore, in the context of SSc, the immune microenvironment undergoes abnormal changes in immune cells, inflammatory cytokines, autoantibodies and extracellular matrix components, ultimately fostering fibrosis and organ damage (28). Consequently, it is imperative to explore the pivotal genes implicated in immune infiltration for the treatment of SSc.

In this study, we initially screened differentially expressed genes (DEGs) of skin samples from early dcSSc patients through analysis of GSE130955 dataset. Subsequently, we employed LASSO regression analysis and the machine learning SVM approach to identify diagnostic genes and construct a diagnostic prediction model. Importantly, we successfully developed a novel diagnostic model, which demonstrated strong predictive power in screening SSc in GSE130955, GSE58095, GSE181549 datasets and our cohort. Furthermore, we validated the differential expression of diagnosis genes in above three cohorts and our cohort. Lastly, we conducted an analysis to explore the potential association between the critical genes and the immune microenvironment of SSc.

## Materials and methods

### Data acquisition and processing

To access the necessary data, we utilized the GEO database maintained by the National Center for Biotechnology Information (NCBI), which offers an open data access platform containing high-throughput sequencing gene expression profiling data. Specifically, we obtained three distinct SSc skin tissue gene expression data from GEO datasets, namely GSE130955, GSE58095, and GSE181549, which were subsequently downloaded for further analysis. GSE130955 encompassed a cohort of 48 early diffuse SSc samples and 33 normal samples, while GSE58095 consisted of 61 SSc samples (dcSSc=43, lcSSc=18) and 36 normal samples, and GSE181549 comprised 113 SSc samples (dcSSc=70, lcSSc=43) and 44 normal samples. In this study, the GSE130955 cohort was utilized as the training cohort, while the GSE58095 and GSE181549 cohorts were employed for the validation purposes.

### Data processing and differentially expressed genes screening

Data processing and screening for DEGs were conducted using the limma package in the R programming language. This involved correcting background errors, normalizing arrays, and performing differential expression analysis on a total of 48 SSc skin samples and 33 normal skin samples in GSE130955. The cutoff values for DEGs were set as having a log fold change (FC) greater than 1 and an adjusted false discovery rate P lower than 0.05.

### Functional enrichment analysis

Utilizing the clusterProfiler software package allowed for the completion of both the Gene Ontology (GO) analysis and the Kyoto Encyclopedia of Genes and Genomes (KEGG) pathway enrichment investigations. This was carried out in order to ascertain the possible activities and pathways in which DEGs are engaged. The outcomes of the study were presented using the visualization module of clusterProfiler since it was the most effective way to do so. In order to carry out disease ontology (DO) enrichment analysis on DEGs, the “clusterProfiler” and DOSE packages found in R were put to use. For the purpose of these studies, a significance level of P 0.05 was used to serve as the cut-off parameter. When comparing the SSc group to the control group, gene set enrichment analysis, also known as GSEA, was employed in order to ascertain which functional terms were more significant than others. We used the “c2.cp.kegg.v7.0.symbols.gmt” file that was found in the Molecular Signatures Database (MSigDB) as the gene set that served as our reference.

### Candidate diagnostic biomarkers

Various machine learning methodologies were explored to identify relevant prognostic markers and generate disease progression forecasts. In the realm of regression analysis, the LASSO approach, which employs regularization to enhance prediction accuracy by reducing the number of exercises in the training set, was employed. The LASSO regression analysis was conducted using the “glmnet” package in R. Additionally, the support vector machine (SVM), a widely recognized supervised machine learning method applicable to both classification and regression tasks, was utilized. To avoid overfitting and ensure accurate results, an RFE approach was employed to select the most suitable genes from the meta-data cohort. Consequently, support vector machine recursive feature elimination (SVM-RFE) was utilized to identify the optimal features for discriminating between different groups. To further validate the expression levels of potential genes, we employed the datasets GSE58095 and GSE181549. The study was expanded to include the overlapping genes that were found as a consequence of employing each of these methodologies simultaneously. In addition, using the predict function that can be found in the glm package of the R programming language, a diagnostic model that was developed using two marker genes was completed. This model was based on the results of the analysis. After that, predictions were created by applying this model to the various types of samples that were contained inside the GSE130955 dataset. In a manner that was comparable to the illustration that came before it, ROC curves were applied for assessing the level of diagnostic ability offered by the novel diagnostic model.

### CIBERSORT-based immune infiltration analysis

The utilization of immune cell deconvolution, a technique based on gene expression profiling, offers a promising alternative to flow cytometry for quantifying immune cells in bulk tissues. Notably, in comparison to flow cytometry, the deconvolution method provides additional details of biological processes and enables retrospective analysis of previous RNA sequencing data available in GEO repositories. Therefore, In this study, the CIBERSORT deconvolution was utilized to quantify the infiltration of 22 immune cells in 48 early SSc skin samples and 33 normal skin samples in GSE130955, facilitating a comparative assessment. To visually represent the distribution of immune cell proportions in each sample, boxplots were generated. The green color denotes the proportion of immune cells in normal samples, while the red color represents the proportion in SSc samples. Furthermore, a nortest analysis, specifically an analysis of variance test, was conducted to evaluate the conformity of the data to a normal distribution. The data were subjected to the t-test method to assess the statistical significance of the results, following confirmation of adherence to a normal distribution. The outcomes of this analysis are presented in the uppermost section of the figure. For correlation analysis and visualization of the 22 distinct immune cell types infiltrating the tissue, the “corrplot” tool in R was employed. Both tasks were executed within the R environment. To depict variations in immune cell infiltration between the SSc and control samples, violin plots were generated using the “vioplot” package in R. These disparities were observed upon comparing the SSc skin samples with the control skin samples.

### Patients and healthy controls

The forearm skin tissue biopsies were obtained from 10 individuals newly diagnosed with dcSSc in accordance with the 2013 criteria established by the American College of Rheumatology (ACR)/European League of Rheumatology (EULAR), including 7 females and 3 males, with a average age of 54.3 ± 9.9 years. Those patients were admitted to the Department of Rheumatology and clinical Immunology at the First Affiliated Hospital of Guangxi Medical University between 2021 and 2023. As a control group, forearm skin tissue biopsies were obtained from 5 individuals of matching age and sex who had undergone internal fixation of forearm fractures for nail removal, including 3 females and 2 males, with a average age of 48 ± 9.03 years. The research protocol adhered to ethical guidelines and was approved by the Ethics Committee of the First Affiliated Hospital of Guangxi Medical University. All participants provided informed consent in writing.

### Real-time quantitative polymerase chain reaction

Isolation of total RNA was achieved using TRIzol Reagent (Invitrogen, Carlsbad, CA, USA). Subsequently, complementary DNA synthesis was performed using the PrimeScriptTM RT Master Mix kit (TaKaRa BIO, Shiga, Japan).Gene expression analysis was conducted using the SYBR Green PCR Kit (TaKaRa) in conjunction with the ABI Prism 7900HT sequence detector. The 2 Ct approach was employed for data analysis, with the GAPDH gene serving as a reference control. Biological replicates were conducted three times. The primer sequences used in our study are listed below: human ENHO forward 5’-CCATTCTCGCTCTGCCGAC-3’, reverse 5’-CAAGCTGGCTAGACTCTGGG’; human NOX4 forward 5’-TGAATCAGATGATGGTCTACACTTG-3’, reverse 5’-AGTGGTCCAAA GGCTTAACATTCC-3’.

### Statistical analysis

All data were processed using R language (version 4.0.2) and statistical analyses were performed using corresponding R package. The unpaired, two-tailed Student’s t-test was employed to assess group differences. Receiver operating characteristic (ROC) analysis was utilized to evaluate the diagnostic relevance of essential genes in SSc samples. Differences were deemed to be significant whenever p was found to be less than 0.05.

## Results

### Identification of DEGs in SSc

**Figure 1:**
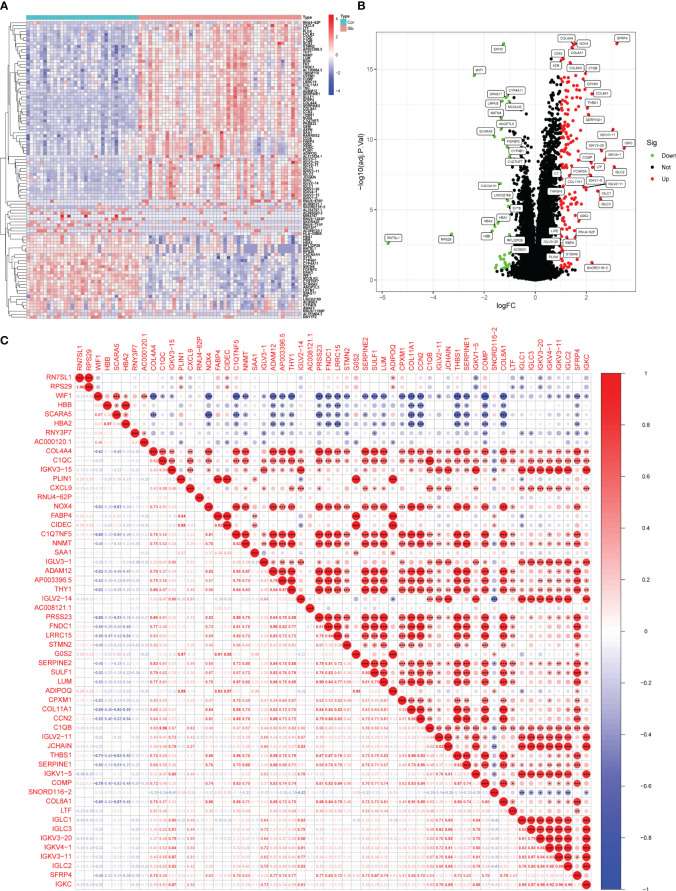
Identification of d DEGs between SSc skin specimens and normal skin specimens. **(A, B)** The expressing pattern of 200 DEGs were shown in Heat map and volcano plot. **(C)** The correlation analysis of 56 DEGs with log FC >1.5. *<0.05, **<0.01, ***<0.001..

In this study, a retrospective analysis was conducted on a dataset (GSE130955) comprising a total of 48 early dcSSc skin samples and 33 normal skin samples. The samples were categorized into SSc and normalgroups, and a comparative analysis was performed. Differential expression analysis was carried out using the limma tool, resulting in the identification of 200 DEGs. Among these DEGs, 163 genes were upregulated, while 37 genes were downregulated (**Figures 1A, B**). Correlation analysis was performed on 56 DEGs with a log fold change (FC) greater than 1.5, as depicted in **Figure 1C**.

### Functional correlation analysis

**Figure 2:**
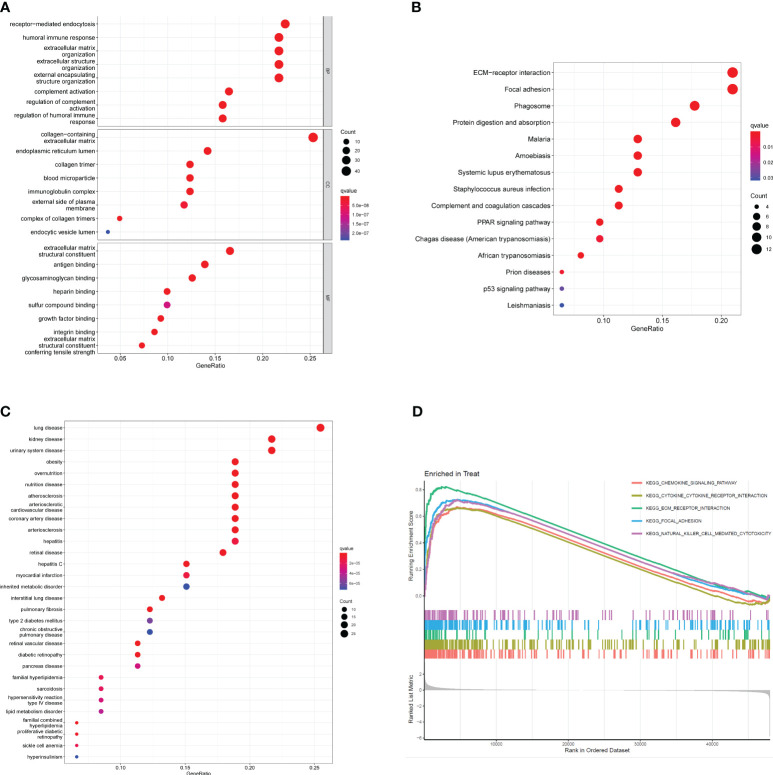
Functional analysis based on 200 DEGs. **(A)** Significantly enriched GO terms of DEGs. **(B)** Significant KEGG pathway terms of DEGs. **(C)** Disease ontology enrichment analysis of 200 DEGs between SSc skin specimens and control skin specimens. **(D)** Enrichment analyses via GSEA.

To investigate the potential functional roles of 200 DEGs in SSc, a functional correlation analysis was conducted. GO analysis revealed that the 200 DEGs were primarily enriched in processes such as receptor-mediated endocytosis, humoral immune response, extracellular matrix organization, endoplasmic reticulum lumen, collagen-containing extracellular matrix, extracellular structure organization, collagen trimer, extracellular matrix structural constituent, antigen binding and glycosaminoglycan binding(**Figure 2A**). Additionally, KEGG analysis indicated that the 200 DEGs were predominantly associated with ECM-receptor interaction, focal adhesion, phagosome, protein digestion and absorption, malaria and amoebiasis (**Figure 2B**). DO enrichment analyses revealed that 200 DEGs were mainly related to lung disease, kidney disease, urinary system disease, obesity, overnutrition and nutrition disease (**Figure 2C**). Subsequently, GSEA assays were conducted to further investigate the enriched pathways in the SSc group. The results demonstrated that the enriched pathways mainly involved chemokine signaling, cytokine-cytokine receptor interaction, ECM receptor interaction, focal adhesion, and natural killer cell-mediated cytotoxicity (**Figure 2D** **)**.

### Identification and validation of diagnostic feature biomarkers

**Figure 3:**
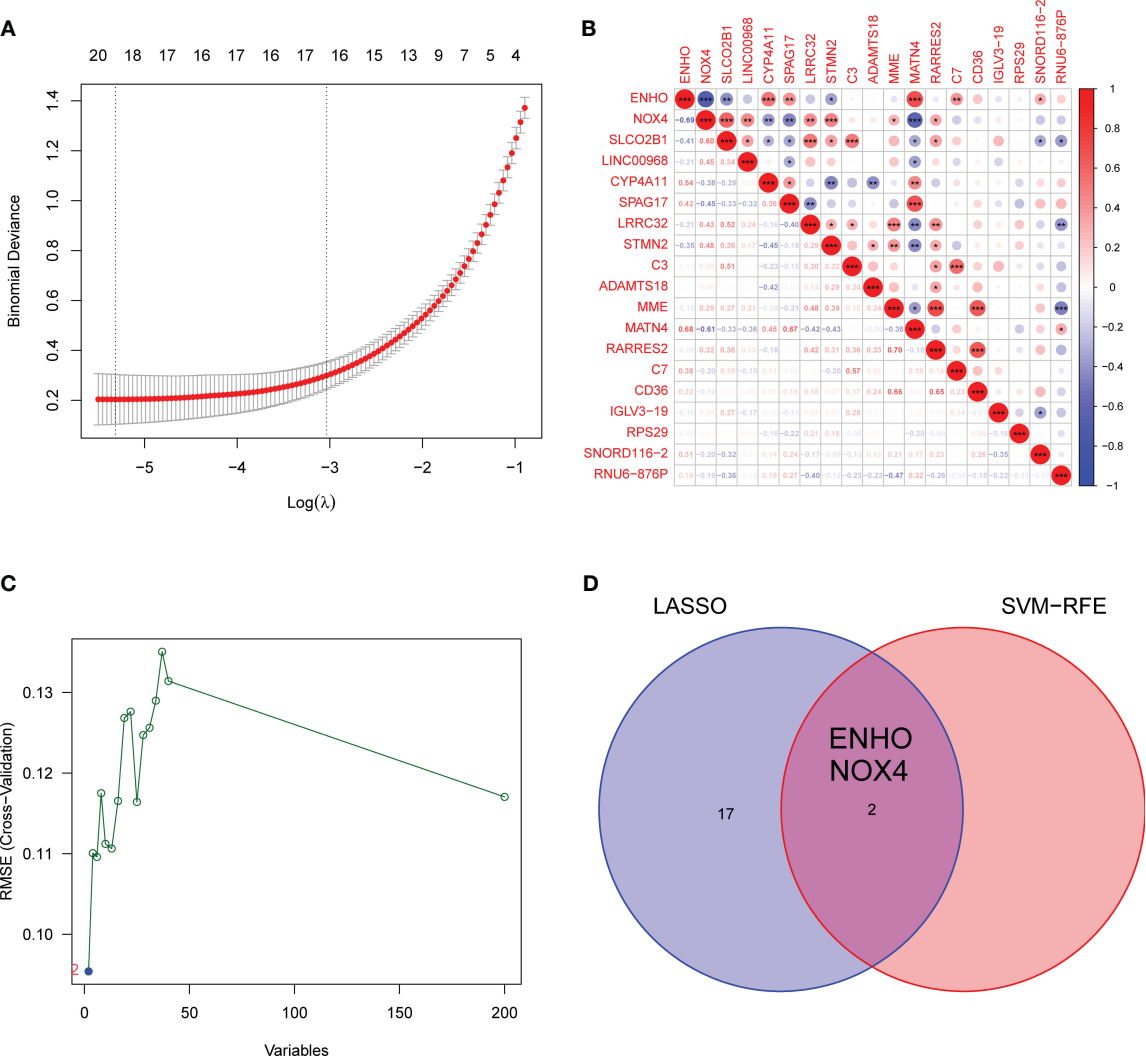
Identification of diagnostic genes for SSc diagnosis. **(A)** LASSO model. **(B)** The correlation analysis of 19 diagnostic genes by LASSO. *<0.05, **<0.01, ***<0.001. **(C)** A plot of biomarkers selection via SVM-RFE methods. **(D)** Venn diagram demonstrating two diagnostic markers (ENHO and NOX4) shared by LASSO and SVM-RFE methods.

**Figure 4:**
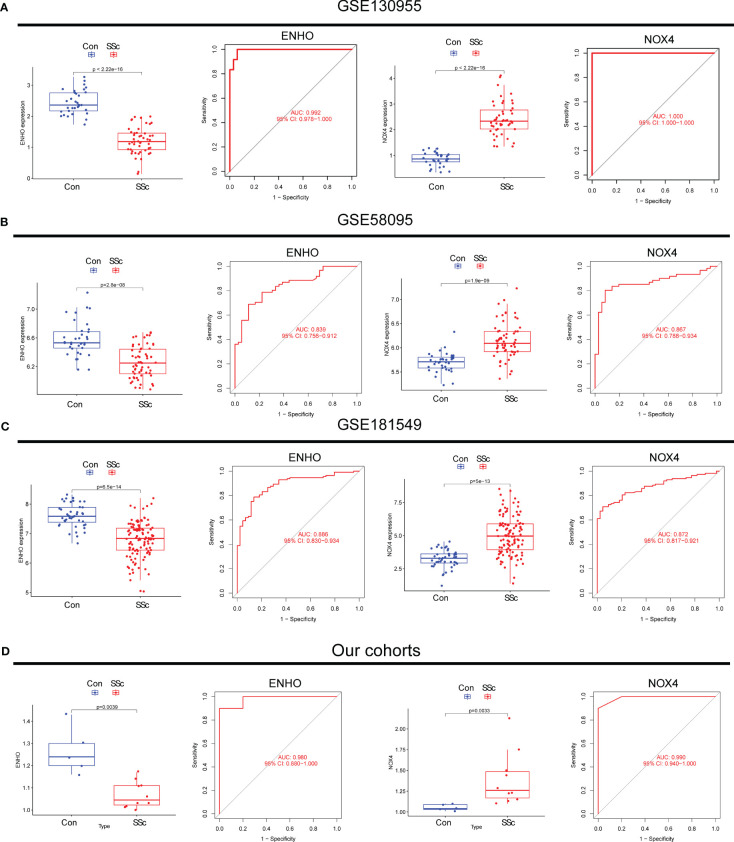
The expression pattern of ENHO and NOX4 and their diagnostic for SSc from **(A)** GSE130955, **(B)** GSE58095, **(C)** GSE181549 datasets and **(D)** our cohorts.

**Figure 5:**
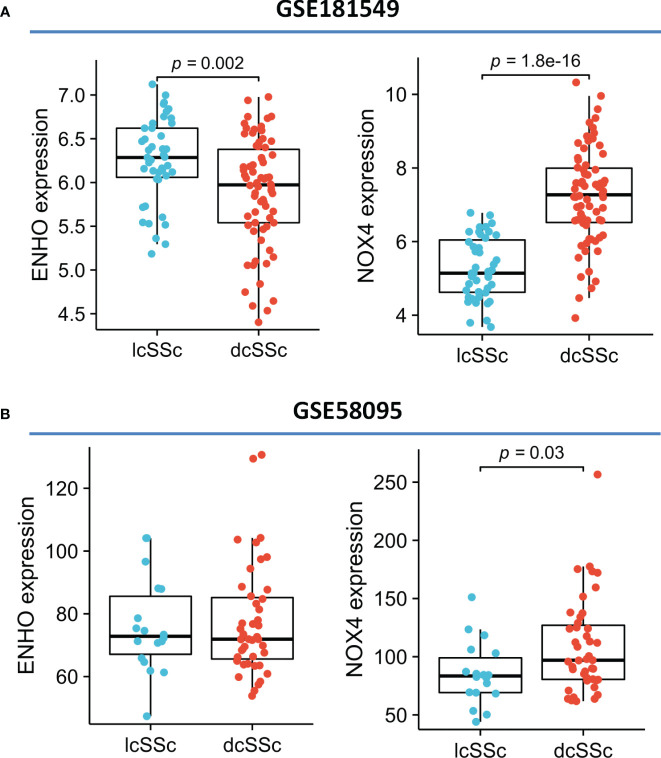
The expression of ENHO and NOX4 in skin specimens of dcSSc and lcSSc from **(A)** GSE181549 and **(B)** GSE58095.

**Figure 6:**
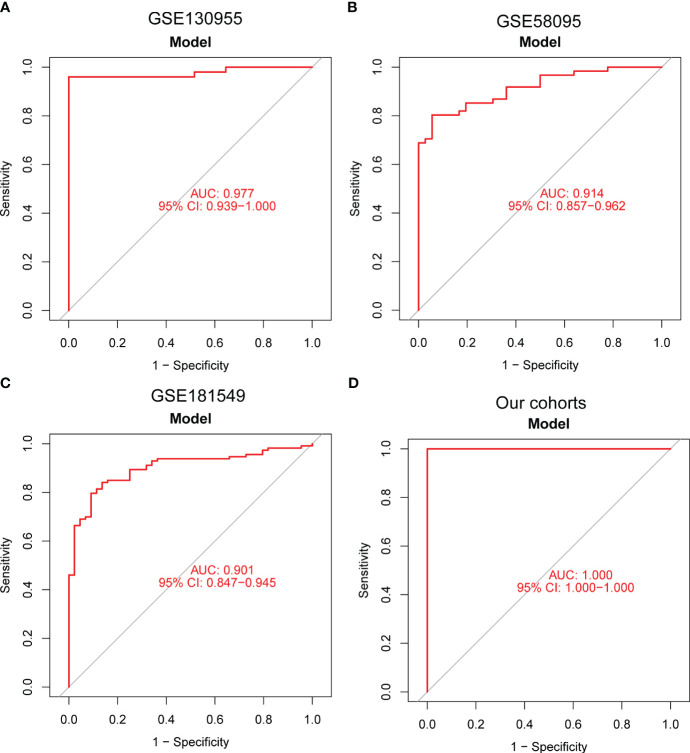
A new diagnostic model was developed using ENHO and NOX4, and its diagnostic value was demonstrated in **(A)** GSE130955, **(B)** GSE58095, **(C)** GSE181549 datasets and **(D)** our cohorts.

Moving forward, the study aimed to identify and validate diagnostic feature biomarkers. Two separate algorithms were employed for this purpose. Firstly, the DEGs were narrowed down using the LASSO regression method, resulting in the identification of 19 potential diagnostic biomarkers for SSc (**Figure 3A**). The association between these 19 genes was analyzed and presented in **Figure 3B**. Additionally, the Support Vector Machine Recursive Feature Elimination (SVM-RFE) methodology was applied, leading to the recognition of a subset of two features that were shared by the DEGs (**Figure 3C**). Ultimately, the two overlapping features, namely ENHO and NOX4, were selected as the final diagnostic biomarkers (**Figure 3D**). Furthermore, the expression and diagnostic value of ENHO and NOX4 were analyzed in the GSE130955, GSE58095 and GSE181549 datasets. ENHO expression levels were found to be significantly decreased in SSc specimens compared to normal specimens. Notably, ENHO demonstrated a strong predictive ability in distinguishing SSc specimens from normal specimens in various datasets, including GSE130955 (**Figure 4A**), GSE58095 (**Figure 4B**), GSE181549 (**Figure 4C**) and our own cohorts (**Figure 4D**). Similarly, we observed that NOX4 expression was markedly increased in SSc samples compared to normal samples. Furthermore, NOX4 exhibited a strong predictive ability in discriminating SSc samples from normal samples in GSE130955 (**Figure 4A**), GSE58095 (**Figure 4B**), GSE181549 (**Figure 4C**) and our own cohorts (**Figure 4D**). Next, we conducted a comparative analysis of ENHO and NOX4 expression levels in the skin of dcSSc and lcSSc subgroups, utilizing data obtained from the GSE58095 and GSE181549 datasets. In the smaller dataset GSE58095 (43 dcSSc *vs* 18 lcSSc), no difference in ENHO expression was observed between two subgroups. However, in the relatively larger dataset GSE181549 (70 dcSSc *vs* 43 lcSSc), a significant decrease in ENHO expression was evident in dcSSc when compared to lcSSc (**Figure 5A**). Conversely, the expression of NOX4 was significantly higher in the skin of dcSSc patients in both datasets, as opposed to lcSSc patients (**Figure 5B**). Subsequently, a diagnostic model was developed utilizing ENHO and NOX4. The diagnostic value of this novel model was further validated in multiple datasets, including GSE130955 (**Figure 6A**), GSE58095 (**Figure 6B**), GSE181549 (**Figure 6C**) and our won cohort (**Figure 6D**), with all AUC>0.7.

### Association between ENHO and NOX4 and CIBERSORT-based immune cells

**Figure 7:**
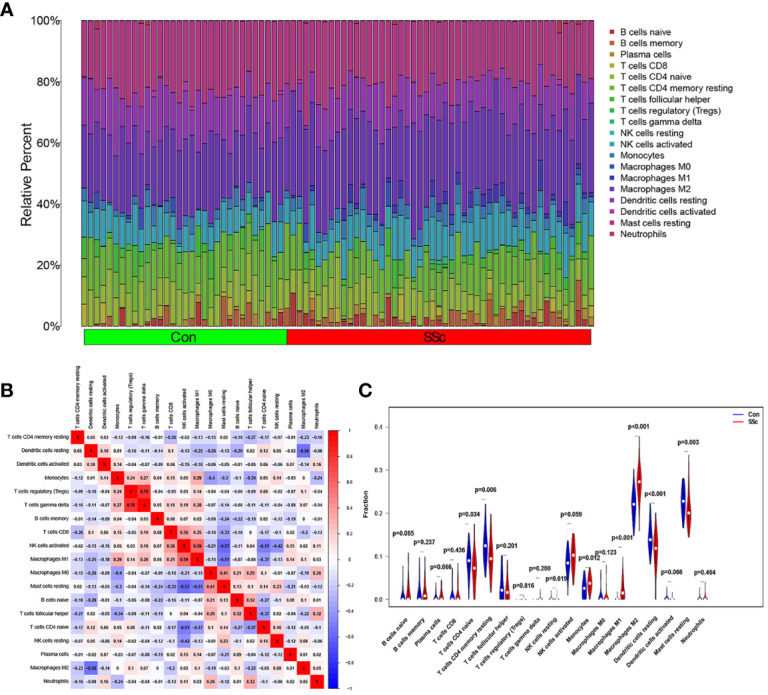
An investigation on the expressions of immune cells and the relationships between them in SSc skin specimens and normal skin specimens was carried out using data from GSE130955. **(A)** Heatmap of 22 immune cells among 48 SSc and 33 normal skin specimens. **(B)** In order to conduct an analysis of the matrix consisting of 22 different types of immune cells, the Pearson correlation coefficient was used. **(C)** The dissimilarity in the composition of immune cells between SSc skin specimens and normal skin specimens.

**Figure 8:**
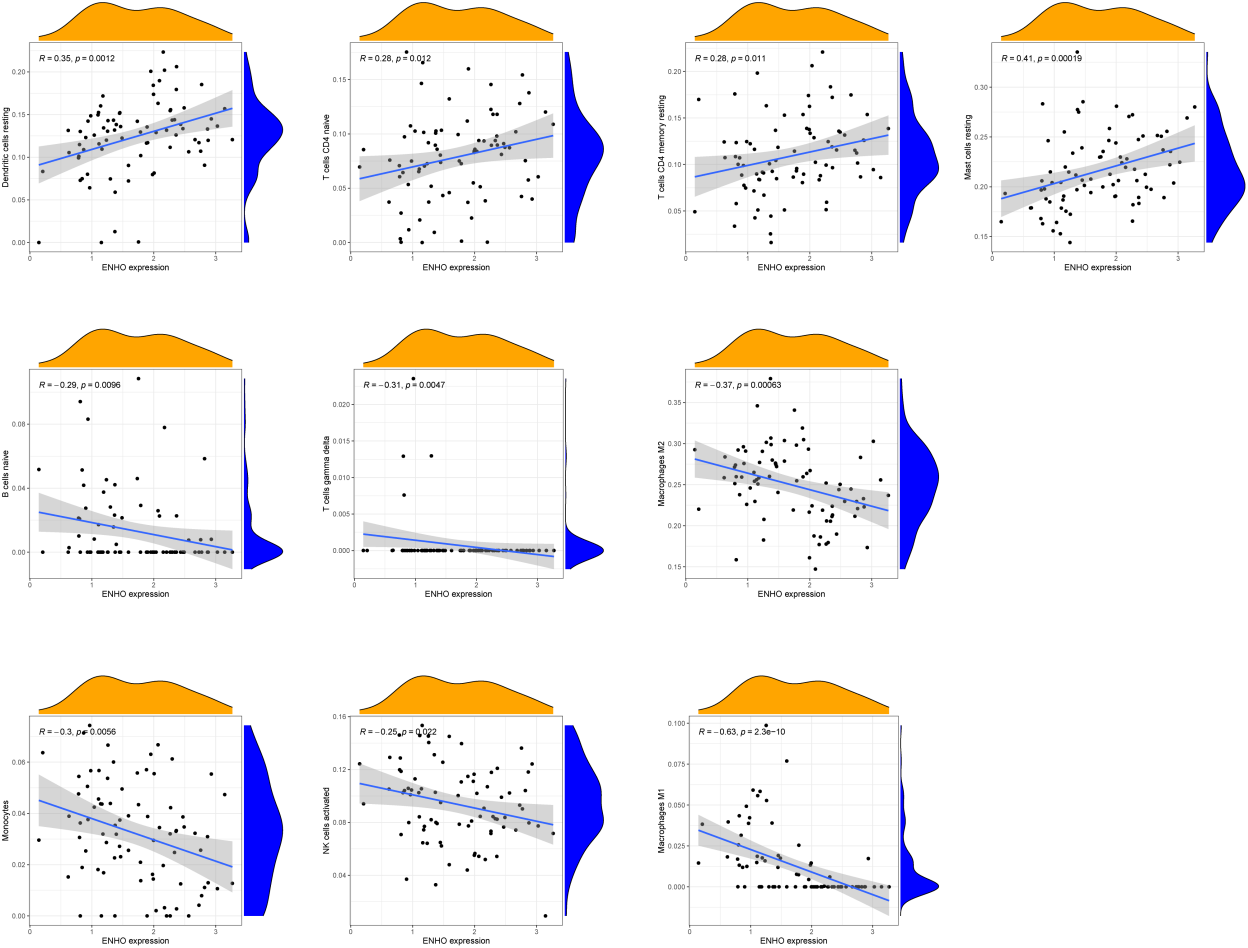
Pearson correlation analysis between NOX4 and immune cells.

**Figure 9:**
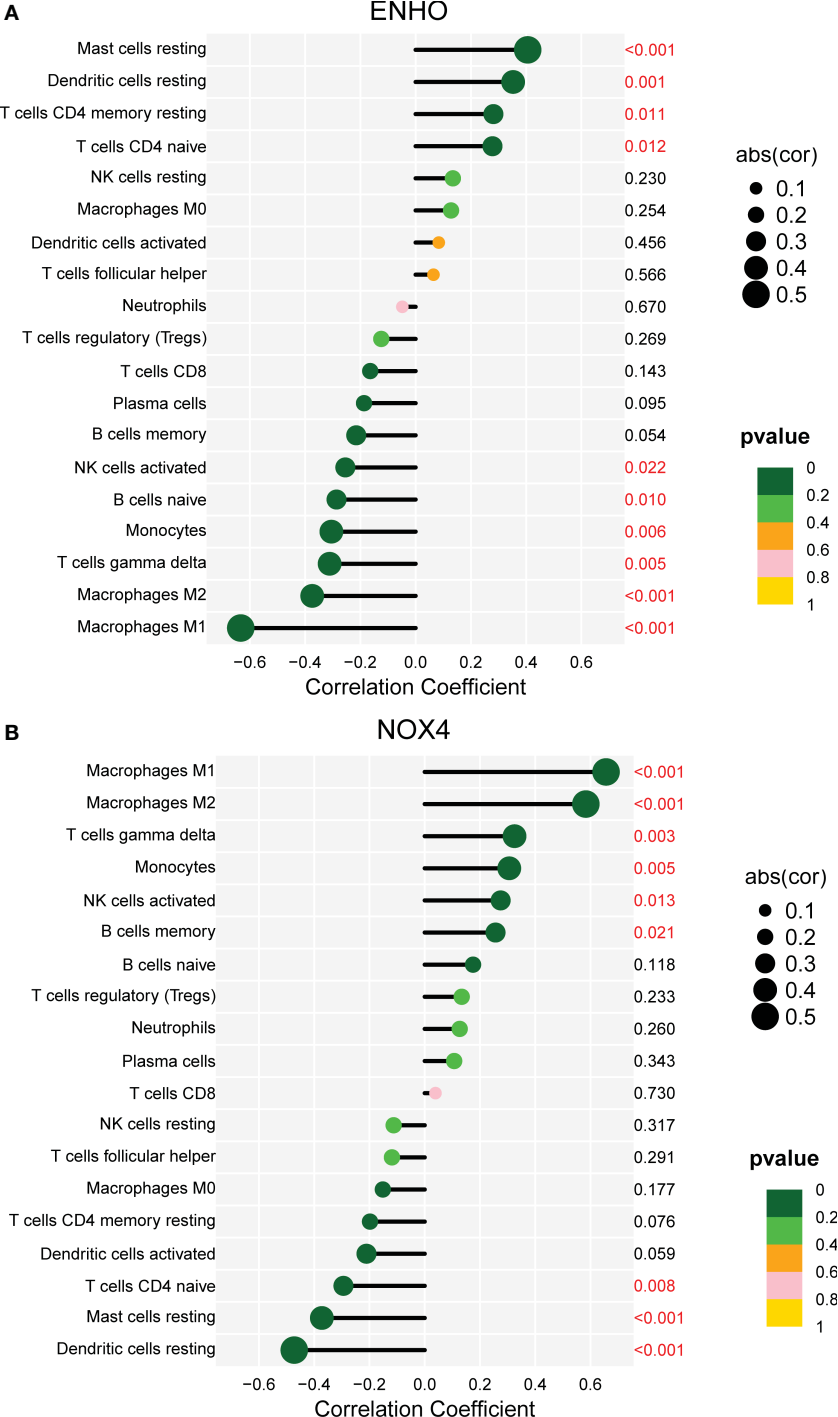
Correlation between ENHO and NOX4, and infiltrating immune cells in SSc skin specimens.

**Figure 10:**
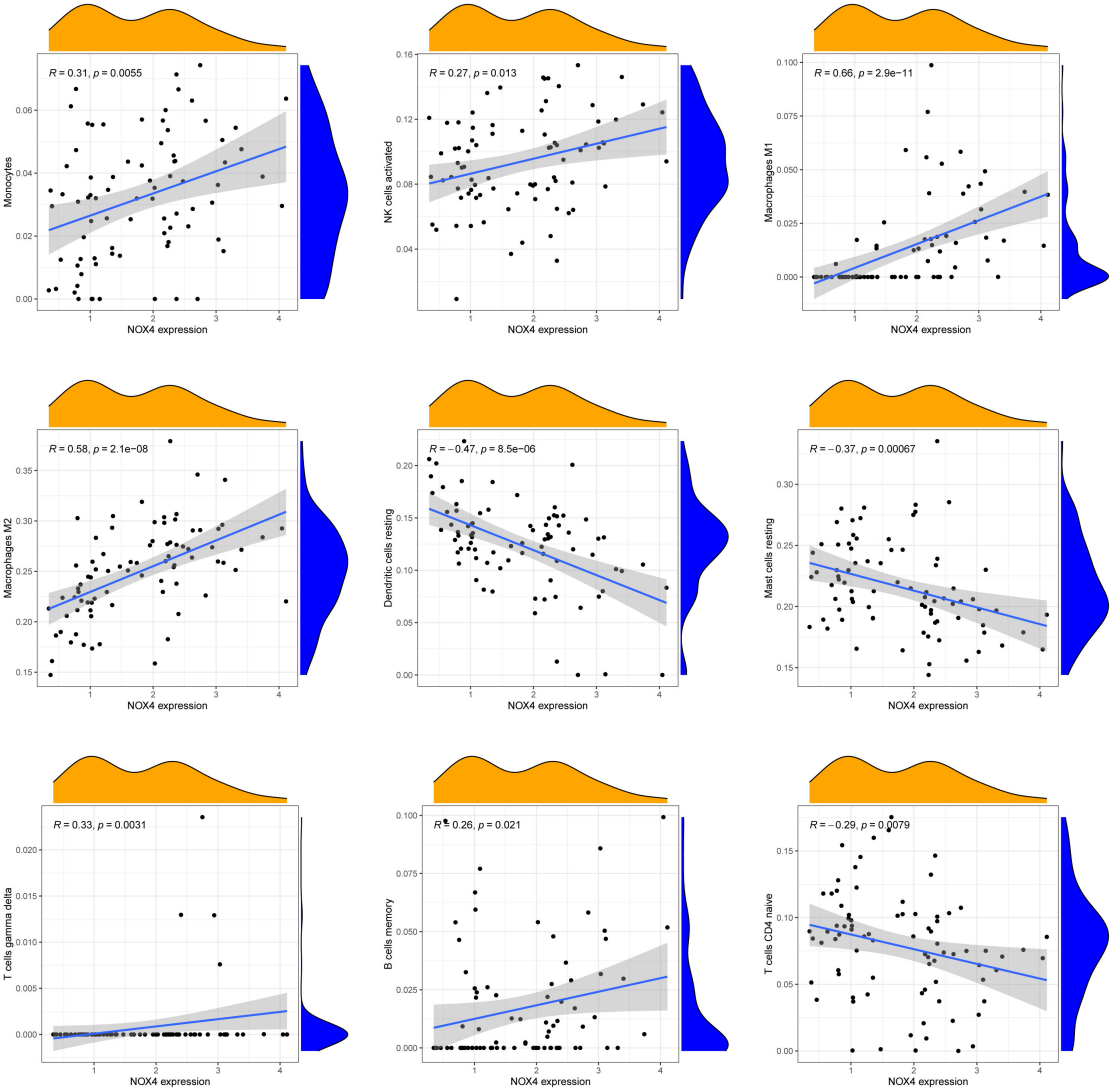
Pearson correlation analysis between ENHO and immune cells.

**Figure 7A** presents a comprehensive visualization of the 22 immune cell subtypes based on CIBERSORT deconvolution that infiltrated in early dcSSc skin specimens and normal skin specimens. Furthermore, we investigated the potential correlations among different immune cell subtypes, as depicted in **Figure 7B**. Significantly, our findings revealed dysregulated levels of various immune cell subtypes within SSc skin specimens. Noteworthy alterations were observed in Monocytes, Macrophages M1, and Macrophages M2, which displayed substantial increases in the skin of individuals with early dcSSc. Conversely, naïve CD4 T cells, resting CD4 memory T cells, resting Dendritic cells, and resting Mast cells exhibited marked reductions in early dcSSc skin when compared to control samples. Furthermore, there was a tendency towards increased levels of naive B cells and plasma cells in SSc, although statistical significance was not observed (**Figure 7C**). Moreover, we conducted an analysis to explore the association between ENHO and NOX4 expression levels and CIBERSORT-based immune cells. Importantly, our results demonstrated a positive correlation between ENHO levels and resting Mast cells, resting Dendritic cells, resting CD4 memory T cells, and naïve CD4 T cells. Conversely, ENHO levels were found to be negatively associated with activated NK cells, naïve B cells, Monocytes, gamma delta T cells, Macrophages M2, and Macrophages M1 (**Figure 8** and **Figure 9A**). Furthermore, our investigation revealed a positive correlation between NOX4 levels and Macrophages M1, Macrophages M2, gamma delta T cells, Monocytes, activated NK cells, and memory B cells. Conversely, NOX4 levels were found to be negatively associated with naïve CD4 T cells, resting Mast cells, and resting Dendritic cells (**Figure 10** and **Figure 9B**).

## Discussion

The therapeutic management of SSc has been challenging, primarily attributed to the complex pathogenesis and delayed detection of the disease. In its initial stages, SSc is characterized by inflammatory processes in affected tissues, which gradually subside as the disease advances, leading to the development of fibrosis in advanced SSc cases (29). Considering the irreversible nature of fibrosis and the promising outcomes observed in several clinical trials where patients with improved skin conditions were predominantly in the inflammatory phase, the identification and early diagnosis of SSc assume paramount importance in the effective management of the disease. Timely recognition enables healthcare practitioners to promptly implement treatment strategies, impede disease progression, attenuate tissue fibrosis, and minimize the risk of organ damage. Notably, recent investigations employing skin transcriptome analysis have unveiled significant profiles of innate and adaptive immune cells in the early stages of SSc (30). Consequently, the identification of specific biomarkers associated with infiltrated immune cells holds promise as a potential method for the early diagnosis of SSc. Further research is warranted to elucidate the precise mechanisms underlying these immune cell infiltrations and to explore their potential as diagnostic markers in the context of SSc.

In this study, we conducted an analysis of the GSE130955 datasets and identified 200 DEGs between SSc skin specimens and normal skin specimens. GO assays were performed to investigate the functional relevance of these DEGs, revealing their close association with humoral immune response and ECM organization and functions. These findings align with the pathological characteristics of SSc, which is characterized by excessive deposition of ECM leading to fibrosis in the skin and internal organs. SSc patients also exhibit humoral immune dysfunction, including B cell activation, increased plasma cells, and overproduction of pathogenic autoantibodies. Notably, some SSc patients have shown positive responses to B cell-depleting therapy (31–33). Furthermore, the GO assays demonstrated a strong correlation between the DEGs and integrin binding to the ECM, which was consistent with the results obtained from the KEGG pathway assays. The KEGG pathway analysis revealed that the DEGs primarily participate in ECM-receptor interaction and focal adhesion. Integrins, known as cell adhesion receptors, play a crucial role in mediating cell attachment to the ECM. Importantly, integrins also activate transforming growth factor-beta (TGF-β) and exert significant effects on the fibrotic process (34, 35). Based on the above findings, the 200 DEGs identified in this study are likely to play crucial roles in the pathogenesis of SSc, encompassing immune dysregulation, extracellular matrix remodeling, cell-cell interactions, as well as metabolism and parasitic infections. Further investigation is warranted to elucidate the specific functions of these genes and their contributions to the underlying mechanisms of the disease.

Furthermore, we employed LASSO and SVM techniques to identify two key genes, namely ENHO and NOX4. ENHO, known as an energy homeostasis-associated gene, is expressed in various tissues including the brain, heart, liver, pancreas, and blood vessels. The transcript of the ENHO gene encodes a secreted protein called adropin (36, 37). Previous studies have reported that ENHO can upregulate the expression of endothelial nitric oxide synthase (eNOS), enhance the release of nitric oxide (NO), improve endothelial cell function, and facilitate neovascularization, thereby exerting a protective effect on the cardiovascular system. Treatment of endothelial cells with adropin has been shown to promote proliferation, migration, and capillary tubule formation, while reducing permeability and tumor necrosis factor-α-induced apoptosis. It has been demonstrated that adropin administration can enhance blood perfusion and increase capillary density in a murine model of hind limb ischemia (38). Notably, reduced levels of adropin in the bloodstream have been associated with endothelial dysfunction (39, 40). Additionally, it has been reported that mutations in ENHO or adropin deficiency can lead to increased expression of vascular endothelial growth factor (VEGF), thereby promoting the proliferation of endothelial cells in both vascular and inflammatory contexts, ultimately triggering MPO-ANCA-associated lung injury (41). Endothelial cell dysfunction and vasculopathy are recognized as characteristic pathological features of SSc, contributing to the development of Raynaud’s phenomenon and ultimately resulting in vascular occlusion and diminished capillary bed. In our investigation, we observed a decrease in ENHO expression in SSc skin tissues, suggesting a potential involvement of ENHO in the pathogenesis of endothelial cell dysfunction and vasculopathy in SSc. Notably, the decreased expression of ENHO in dcSSc skin tissue is more pronounced than that in lcSSc, suggesting that ENHO may be associated with the skin severity of SSc. Furthermore, Servet et al. reported a decrease in ENHO expression specifically in SSc patients with lung involvement compared to those without, while no significant difference in ENHO mRNA expression was observed in peripheral blood mononuclear cells (PBMCs) of SSc patients compared to normal controls (42). These findings and our results suggest that ENHO acts primarily in the tissue lesions of SSc rather than in the peripheral blood. This highlights the need for further investigation into the precise role of ENHO in the context of SSc.

NOX4, a gene encoding the enzyme NADPH oxidase 4, is highly expressed in fibroblasts, microvascular endothelial cells, lung epithelial cells, kidney and smooth muscle cells (43). Studies have shown that NOX4 were upregulated in SSc dermal fibroblasts. NOX4 plays a crucial role in the generation of intracellular reactive oxygen species (ROS), NOX4-stimulated ROS production may be involved in the development of tissue fibrosis, abnormalities of endothelial and vascular, and even the generation of autoantibodies. Reduction or abrogation of NOX4 decreased ROS production and expression of type I collagen and α-smooth muscle actin in SSc fibroblasts (43–46). Moreover, TGF-β, a well-recognized profibrotic cytokine, has been demonstrated to promote the expression of NOX4 in dermal and pulmonary fibroblasts. Deletion of Nox4 abolished TGF-β1-induced fibrosis in the skin and lung tissues of mice (47). Our results further confirmed that NOX4 expression was significantly increased in SSc skin specimens, especially in patients with dcSSc, suggesting that NOX4 may contribute to the progression of skin lesions in SSc. Taken together, these findings suggest that NOX4 may represent a promising therapeutic target for SSc.

Building upon these observations, we have developed a novel diagnostic prediction model utilizing ENHO and NOX4, and its diagnostic predictive efficacy has been further validated in multiple datasets (GSE130955, GSE58095, GSE181549) as well as our own cohort. This finding, based on the novel diagnostic prediction model incorporating ENHO and NOX4, has demonstrated its predictive power across various datasets and our cohort, indicating its role in early detection and prediction of SSc risk. Lafyatis et al. previously developed a four-gene biomarker for predicting skin disease in dcSSc, including two TGFβ-regulated genes (cartilage oligomeric matrix protein, COMP and thrombospondin-1, THS1) and two interferon(IFN)-regulated genes(interferon-induced 44, IFI44 and sialoadhesin, SIG1). However, it is important to note that their approach differs from our study. They utilized RT-PCR to measure the expression levels of known TGFβ and IFN-regulated genes, and subsequently correlated these levels with the modified Rodnan skin score (MRSS) using multiple regression analyses to achieve the best-fit models. The four-gene predictor serve as an objective surrogate outcome measure, complementing the skin score evaluations in dcSSc (48)**.**

The immune microenvironment plays a crucial role in the etiology and progression of SSc. A comprehensive understanding of the interplay between the immune microenvironment and SSc can provide valuable insights into the disease’s pathogenesis and facilitate the development of therapeutic interventions. Our analysis of immune infiltration using CIBERSORT deconvolution revealed an elevated presence of Macrophages M1 and Macrophages M2 in the skin of early dcSSc patients, which aligns with previous research findings (49, 50). Macrophages exhibit remarkable plasticity and can undergo distinct forms of polarized activation, traditionally classified as M1 (classically activated) and M2 (alternatively activated) macrophages. M1 macrophages contribute to inflammatory processes that are detrimental to overall health and can lead to tissue damage, suggesting their potential involvement in the early stage of SSc. Conversely, M2 macrophages release profibrotic factors such as TGF-β and contribute to the development of fibrosis in the later stages of the disease (51, 52). Interestingly, we observed a negative correlation between the levels of ENHO and Macrophages M1 and M2, while the levels of NOX4 showed a positive association with Macrophages M1 and M2. The latest findings have demonstrated that adropin promotes M2 polarization of lung macrophages and mitigate the severity of pancreatitis‐associated lung injury (53). Adropin deficiency has been shown to result in increased infiltration of M1 macrophages in colon and mesentery tissues, leading to the development of colitis. Additionally, an imbalance of M2 to M1 macrophage polarization was observed in colon and mesentery of Enho-/- mice (54). These studies indicate the potential of ENHO to regulate macrophage polarization, although its role in SSc macrophages remains unexplored. Notably, pulmonary macrophages from individuals with asbestosis-induced pulmonary fibrosis exhibit elevated expression of NOX4, which contributes to apoptosis resistance in monocyte-derived macrophages and the progression of pulmonary fibrosis (55). Furthermore, NOX4 has been implicated in the regulation of macrophage polarization through various mechanisms (56, 57). However, the role of NOX4 in SSc macrophages has yet to be investigated. Our findings suggest that ENHO and NOX4 may potentially promote the progression of SSc by exerting negative and positive regulatory effects on Macrophages M1 and M2, respectively. Further researches are necessary to fully elucidate these mechanisms.

## Conclusion

Following LASSO and SVM analysis, we have successfully identified two pivotal genes, ENHO and NOX4, that play a crucial role in the pathogenesis and progression of SSc. To validate our findings, we conducted extensive analyses on multiple datasets as well as our own SSc cohorts. As a result, we have developed a novel diagnostic model utilizing ENHO and NOX4 as biomarkers, which exhibits promising clinical utility in terms of early diagnosis and disease risk prediction. Notably, ENHO and NOX4 have been found to be associated with immune cell activation and fibroblast activity. Moreover, our study suggests that ENHO and NOX4 may contribute to the advancement of SSc by exerting negative and positive regulatory effects on macrophages M1 and M2, respectively. In conclusion, this study provides valuable insights into the potential clinical significance of ENHO and NOX4 in early diagnosis and risk prediction of SSc. However, further clinical investigation is warranted to evaluate the efficacy and feasibility of implementing this model in real-world clinical settings.

## Data availability statement

The datasets presented in this study can be found in online repositories. The names of the repository/repositories and accession number(s) can be found in the article.

## Ethics statement

The studies involving humans were approved by the Ethics Committee of the First Affiliated Hospital of Guangxi Medical University. The studies were conducted in accordance with the local legislation and institutional requirements. The participants provided their written informed consent to participate in this study.

## Author contributions

LZ: Methodology, Investigation, Writing – original draft. QW: Investigation, Visualization, Writing – review & editing. SC: Validation, Formal analysis, Writing – review & editing. JW: Validation, Writing – review & editing. FD: Formal analysis, Writing – review & editing. NM: Data curation, Writing – review & editing. WZ: Project administration, Writing – review & editing. CZ: Writing – review & editing. XZ: Conceptualization, Writing – review & editing.
